# Prevalence and temporal trends of physical activity counselling in primary health care in Germany from 1997–1999 to 2008–2011

**DOI:** 10.1186/s12966-015-0299-9

**Published:** 2015-10-26

**Authors:** Lars Gabrys, Susanne Jordan, Martin Schlaud

**Affiliations:** Robert Koch Institute – Department of Epidemiology and Health Monitoring, General Pape Str. 62-66, 12101 Berlin, Germany

**Keywords:** Physical activity, Health promotion, Counselling, Prevention, Epidemiology

## Abstract

**Background:**

For patients, usually the first and most preferred contact person on health issues is still the doctor and most persons see their doctor at least once a year. Therefore, physical activity counselling strategies delivered by a physician seem to be a promising approach for physical activity improvement. The aim of this work is to show prevalence and time trends in physical activity counselling by primary health care physicians from 1997–1999 to 2008–2011 in Germany.

**Methods:**

Data from two representative cross-sectional health interview and examination surveys of the Robert Koch Institute were used. Prevalence proportions of physicians’ physical activity counselling and patients’ utilisation of health promotion programmes in relation to physical activity counselling were analysed. Strengths of associations were calculated by using binary logistic regression models. Overall, 11,907 persons aged 18–64 years were included in the analyses.

**Results:**

Physical activity counselling prevalence decreased from 11.1 to 9.4 % in men and from 9.3 to 7.7 % in women over ten years. Only persons with accumulated health risks (OR 5.33; 95 % CI 1.89–15.00) and persons with diagnosed diabetes mellitus (OR 3.42; 95 % CI 1.68–6.69) showed significantly higher counselling proportions in 2008–2011 compared to 1997–1999. Men were more often counselled on physical activity than women, but women showed significantly higher participation rates in physical activity promotion programmes in both surveys. In both sexes significantly higher participation rates could be observed in persons who had received some activity counselling by a physician.

**Conclusion:**

Although, evidence underlines the positive health effects of regular physical activity; overall, physicians counselling behaviour on physical activity decreased over time. However, it is positive to note that a trend towards a disease-specific counselling behaviour in terms of a tailored intervention could be observed.

## Background

In 2013, Joy et al. published an article entitled *physical activity counselling in sports medicine: a call to action* [1]. Therein they stated the enormous potential of physical activity for the prevention and management of most chronic non-communicable diseases (NCDs), and as they call it the ethical and legal obligation of physicians to encourage and help their patients to be more physically active.

Physical activity levels according to the guidelines can potentially lower the risk of cardiorespiratory diseases by 35 % and of type 2 diabetes or metabolic syndrome by 30–40 %, compared to sedentary individuals, and overall mortality can be reduced by 32 % when comparing most with least active subjects [2, 3]. Additionally, physical activity and exercise are associated with decreased risks of mental disorders like depression or anxiety [4, 5], improved muscular and bone health [6, 7], reduced cancer rates [8] as well as an overall increase in cardiorespiratory fitness and wellbeing [3]. Furthermore, physical inactivity is held responsible for 4.2–19.2 % of all-cause mortality within the European Union and about 7.5 % in Germany [9].

Beside the growing body of evidence regarding the positive health effects of regular physical activity and the integration of physical activity recommendations in national and international guidelines [2, 8, 10], quality assured prevention programmes as well as structured disease management programmes for many NCDs were developed and implemented in the German health care system in the year 2000 and 2003 respectively [11]. Next to pharmacological therapies, lifestyle modifications including physical activity improvements are major concepts of many such programmes. For patients, usually the first and most preferred contact person on health issues is still the doctor and most persons see their doctor at least once a year [13, 14]. Therefore, physical activity counselling strategies delivered by physicians seem to be a promising public health approach for physical activity improvements on a population-wide level. Close connections to providers of prevention and disease management programmes may improve patient-centred care and may help to reduce costs in the health care system. Barnes et al. [15] showed time trends from 2000 to 2010 of physical activity counselling rates in the U.S. They reported an overall increase of about 10 % in U.S. adults who had received physician’s or other health professional’s advice to exercise or other physical activity in the last 12 months [15].

Addressing these facts, it was of particular interest if physicians’ counselling behaviour changed over time and if physical activity counselling is associated with patients’ utilisation of health promotion measures. Therefore, the aim of this work is to examine prevalences and temporal trends in physical activity counselling by primary health care physicians and participation rates in physical activity promotion programmes from 1997–1999 to 2008–2011 in Germany.

## Methods

### Dataset

Two representative health surveys of the Robert Koch Institute were used to determine physical activity counselling prevalences and to calculate temporal trends. The first “German National Health Interview and Examination Survey 1998” (GNHIES 98) was conducted from 1997 to 1999 and the “German Health Interview and Examination Survey for Adults” (DEGS 1) was conducted from 2008 to 2011. Both surveys are part of the ongoing health monitoring system of the Robert Koch Institute. The recruitment strategies of GNHIES 98 and DEGS 1 consisted of age- and sex-stratified random samples selected from local population registries; participants of the GNHIES 98 were repeatedly invited to participate in DEGS 1. Methods, study design and exact study compositions are described elsewhere [16, 17]. Overall, study population of GNHIES 98 consists of 7124 persons and DEGS 1 consists of 8152 persons between 18 and 79 years of age. Participation in both surveys was voluntary and informed consent was obtained from all participants. Both surveys were approved by the Charité Universitätsmedizin Berlin ethics committee and by the Federal Commissioner for Data Protection.

In addition to an extensive health examination programme, both surveys comprised a self-administered questionnaire on different health issues like current or former (chronic) diseases, individual health behaviour like diet or physical activity, socio demographic and socio economic parameters and health care utilisation as well as participation in prevention programmes. To answer the research question of this paper, our main focus was on the data on self-reported physical activity counselling provided by a primary health care physician over the last 12 months before the interview. Because this question was only asked to persons up to 64 years of age, participants aged 65 and above were excluded from the analyses.

### Data analyses

Stratified analyses of men and women as well as analyses specific for disease (coronary heart disease, hypertension, diabetes, any type of cancer), socio demographic and socio economic factors (BMI, socio economic status, duration of sport participation per week) were conducted with data from both surveys separately. Socio economic status (SES) is based on educational background, occupational status and income. The upper and the lowest 20 % of the sample were classified as high SES and low SES respectively; the remaining 60 % were assigned as middle SES [18]. Stratified trend analyses were conducted between 1997–1999 and 2008–2011, but not gender specific. For a more detailed and in-depth analysis of physicians physical activity counselling behaviour, risk scores (0–3) for accumulated cardio-metabolic health risks were calculated for each participant. Every predefined cardiovascular risk factor (diabetes, overweight, hypertension) was assigned with a score-value of 1. Risk scores of 0–1 were classified as normal cardio-metabolic risk; score-values of 2 as elevated risk and score-values of 3 as high cardio-metabolic risk. Additionally, stratified participation rates in physical activity promotion programmes in association with physical activity counselling were analysed and time trends were calculated.

To obtain reliable and comparable outcomes, design weights were calculated, regarding socio economical characteristics, such as age and sex. In a second step the overall sample was adjusted to the population figures of the Federal Statistical Office for the year 2010 [17]. All statistical analyses were conducted by using SAS 9.4. Univariate logistic regression models for complex samples (PROC SURVEYLOGISTIC) were applied to calculate stratified effect measures and trend analyses.

## Results

After excluding participants’ aged 65 years or older, 11,907 persons remained in the final dataset. Exact Information on the age distributions can be seen in Table 1.

**Table 1:** Absolute numbers of men and women in GNHIES 98 and DEGS 1

	GNHIES 98 (1997–1999)		DEGS 1 (2008–2011)	
Age [years]	Men	Women	Men	Women
18–29	626	636	525	547
30–39	731	785	473	541
40–49	606	671	716	823
50–59	645	677	735	857
60–64	283	309	340	381
Total	2891	3078	2789	3149

**Fig. 1:**
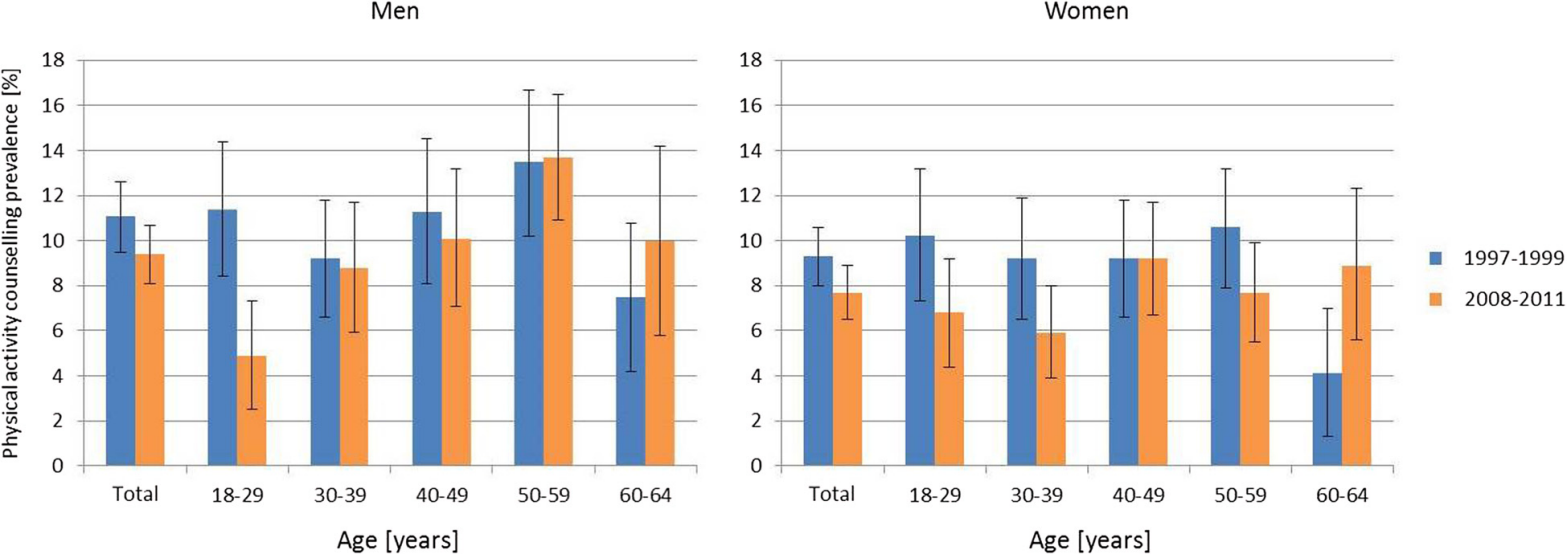
Age stratified prevalence estimates of physicians’ counselling on patients’ physical activity behaviour. Detailed legend: Prevalence estimates incl. 95 % confidence intervals are shown for the time periods of both health surveys; 1997–1999 (GNHIES 98) and 2008–2011 (DEGS 1)

As shown in Fig. 1, the counselling prevalence decreased from 1997–1999 to 2008–2011 in both sexes in almost all age groups. In 2008–2011, only persons aged 60 years or older reported a higher counselling prevalence compared to the earlier time period. In men, a continuous increase in self-reported physical activity counselling from younger to older age groups with a peak in men 50–59 years could be observed in both surveys with an exception in 18–29 year old men in 1997–1999. After the age of 60, the counselling prevalence seemed to decrease again to a lower level. Although, it is important to note that these observations are only statistically significant in the age group 18–29 years in men. In women this trend towards increasing counselling rates by age could not be observed. The analyses showed slightly but not significantly higher physical activity counselling proportions in men compared to women in almost all age groups (Fig. 1).

In the time period of 2008–2011, physical activity counselling prevalence was significantly higher in obese persons than in non-obese (men: OR 2.56, 95 % CI 1.67–3.93; women: OR 1.62, 95 % CI 1.08–2.42). This was also true for diabetes mellitus (men: OR 4.51, 95 % CI 2.54–8.01; women: OR 4.67, 95 % CI 2.65–8.25), hypertension (men: OR 2.29, 95 % CI 1.64–3.21; women: OR 1.90, 95 % CI 1.30–2.79) and coronary heart disease (OR 2.56, 95 % CI 1.54–4.27). However, these condition-specific differences were not seen in 1997-1999. The total number of cancer patients was too small for reliable effect estimates. Higher counselling proportions towards persons who were more physically active were only seen in the time period 1997–1999, but not in 2008–2011. In both time periods a social gradient towards higher counselling prevalences in the highest socio economic group can be observed (Table 2).

**Table 2:** Temporal trends in prevalence of physical activity counselling and effect estimates of stratified group comparisons

Physical activity counselling prevalence									
	1997–1999				2008–2011				1997–1999 vs 2008–2011
	(%)		OR (95 % CI)		(%)		OR (95 % CI)		OR (95 % CI)
Men	11.1		Ref.		9.4		Ref.		0.84 (0.62–1.03)
Women	9.3		0.82 (0.67–1.02)		7.7		0.80 (0.63–1.02)		0.82 (0.65–1.02)
		Men		Women		Men		Women	Total
	(%)	OR (95 % CI)	(%)	OR (95 % CI)	(%)	OR (95 % CI)	(%)	OR (95 % CI)	OR (95 % CI)
BMI									
18.5 – <25.0	9.8	Ref.	9.4	Ref.	6.5	Ref.	6.2	Ref.	0.64*** (0.53–0.77)
25.0 – <30.0	11.4	1.19 (0.86–1.63)	10.2	1.10 (0.81–1.49)	8.8	1.39 (0.94–2.08)	6.5	1.06 (0.73–1.53)	0.70** (0.54–0.91)
30.0 +	12.2	1.29 (0.84–1.96)	7.8	0.82 (0.54–1.25)	15.1	2.56*** (1.67–3.93)	9.7	1.62* (1.08–2.42)	1.30 (0.92–1.82)
Diabetes									
No	11.1	Ref.	9.2	Ref.	8.6	Ref.	7.1	Ref.	0.76*** (0.65–0.88)
Yes	10.8	0.97 (0.46–2.03)	9.8	1.08 (0.39–2.99)	29.8	4.51*** (2.54–8.01)	26.4	4.67*** (2.65–8.25)	3.42*** (1.68–6.96)
Coronary heart disease									
No	10.8	Ref.	9.2	Ref.	10.9	Ref.	8.5	Ref.	0.97 (0.80–1.17)
Yes	17.7	1.77 (0.95–3.29)	9.2	1.01 (0.40–2.53)	24.0	2.56*** (1.54–4.27)	7.6	0.89 (0.24–3.26)	1.36 (0.68–2.72)
Hypertension									
No	10.6	Ref.	9.3	Ref.	8.1	Ref.	6.9	Ref.	0.73*** (0.62–0.86)
Yes	14.1	1.39 (0.93–2.07)	8.4	0.89 (0.58–1.36)	16.8	2.29*** (1.64–3.21)	12.3	1.90** (1.30–2.79)	1.36 (0.97–1.90)
Cancer									
No	11.1	Ref.	9.1	Ref.	9.3	Ref.	7.6	Ref.	0.82* (0.71–0.96)
Yes	9.0	0.79 (0.24–2.58)	10.2	1.13 (0.45–2.83)	14.0	1.59 (0.74–3.39)	8.2	1.08 (0.54–2.19)	1.05 (0.44–2.51)
Socio economic status (SES)									
Low	9.0	Ref.	7.7	Ref.	5.9	Ref.	6.4	Ref.	0.72 (0.47–1.11)
middle	9.8	1.10 (0.70–1.72)	8.1	1.06 (0.70–1.62)	9.4	1.67 (0.93–2.98)	7.9	1.25 (0.75–2.08)	0.96 (0.79–1.18)
High	15.6	1.86* (1.15–2.99)	14.4	2.03** (1.23–3.35)	12.0	2.18** (1.21–3.95)	8.1	1.28 (0.70–2.33)	0.64** (0.47–0.87)
Sports activity									
No sports	7.7	Ref.	5.4	Ref.	7.5	Ref.	7.2	Ref.	1.15 (0.85–1.55)
<1 h/week	13.1	1.82* (1.14–2.89)	11.4	2.28*** (1.45–3.60)	10.7	1.49 (0.94–2.55)	6.4	0.89 (0.55–1.45)	0.67* (0.47–0.95)
1–2 h/week	13.8	1.93** (1.29–2.88)	11.2	2.23*** (1.45–3.41)	11.1	1.55 (0.94–2.55)	10.3	1.47* (1.03–2.12)	0.85 (0.62–1.17)
2–4 h/week	9.7	1.29 (0.83–2.00)	14.8	3.06*** (2.02–4.65)	10.3	1.42 (0.83–2.42)	6.5	0.89 (0.56–1.41)	0.67* (0.48–0.94)
> 4 h/week	17.8	2.61*** (1.75–3.90)	17.1	3.65*** (1.99–6.68)	8.1	1.09 (0.63–1.88)	6.1	0.84 (0.46–1.51)	0.38*** (0.25–0.58)
Cardio-metabolic risk (score)									
normal risk (0–1)	10.5	Ref.	9.3	Ref.	7.8	Ref.	7.0	Ref.	0.73*** (0.62–0.85)
elevated risk (2)	15.8	1.60* (1.07–2.40)	9.1	0.98 (0.60–1.58)	18.0	2.60*** (1.84–3.67)	11.4	1.72* (1.08–2.74)	1.22 (0.83–1.79)
high risk (3)	5.1	0.46 (0.13–1.65)	11.1	1.22 (0.39–3.85)	31.2	5.37*** (2.42–11.89)	33.1	6.62*** (2.76–15.84)	5.33*** (1.89–15.00)

*p < 0.05; **p < 0.01; ***p < 0.001

When looking at temporal trends between 1997–1999 and 2008–2011 (Table 2), a major decrease in the prevalence of physical activity counselling can be seen. Only persons with diagnosed diabetes mellitus (OR 3.42, 95 % CI 1.68–6.96) and persons in the highest risk group for cardio-metabolic diseases (OR 5.33, 95 % CI 1.89–15.00) reported significantly higher physical activity counselling proportions in the later than in the earlier time period. Thus, the counselling prevalence in diabetic men increased by 19 % and in diabetic women by 16.6 %. Men with accumulated cardio-metabolic health risks reported an increase by 26.1 % and women by 22.0 % from 1997–1999 to 2008–2011. Persons with a high risk-score were almost 6-fold more likely to have received physical activity counselling by their primary health care physicians than were persons in the lowest risk-group in 2008–2011 (OR 5.88, 95 % CI 3.18–10.87).

Self-reported participation in physical activity promotion programmes was significantly more frequent in women than in men, regardless of whether or not they had received some physical activity counselling. Men and women who had received some counselling were significantly more likely to have participated in physical activity promotion programmes. Overall, temporal trends regarding participation in physical activity promotion programmes showed a significant increase in both sexes from 1997–1999 to 2008–2011. All effect measures and prevalence estimates are listed in Table 3.

**Table 3:** Temporal trends in prevalence of participation in physical activity programmes in accordance to physical activity counselling and effect estimates of group comparisons

	1997–1999			2008–2011			1997–1999 vs. 2008–2011
	Counselling	No counselling	OR (95 % CI)	Counselling	No counselling	OR (95 % CI)	OR (95 % CI)
Men	10.0	5.3	2.00** (1.22–3.30)	18.6	7.5	2.82*** (1.85–4.27)	1.57*** (1.21–2.06)
Women	20.8	9.5	2.50*** (1.65–3.78)	31.7	17.5	2.20*** (1.58–3.05)	2.02*** (1.68–2.44)
OR (95 % CI)	2.40** (1.29–4.33)	1.89*** (1.47–2.44)		2.04** (1.30–3.18)	2.61*** (2.11–4.21)		

*p < 0.05; **p < 0.01; ***p < 0.001

## Discussion

This paper analysed prevalences of physical activity counselling by primary health care physicians during consultations on the one hand and patients’ participation in physical activity promotion programmes on the other hand, with special emphasis on temporal trends between the study periods 1997–1999 and 2008–2011 in Germany.

In both surveys every effort was made to obtain representative random samples from the general population. Slightly higher response proportions of elderly people were controlled for by applying survey weights in data analyses. All non-participants were asked to complete a non-responder questionnaire, which was answered by 42 % of non-participants. Comparisons between participants and non-participants did not show any substantial differences [16, 17]. Therefore, we assume that any conclusions drawn from the samples can be generalised to the German population. Since the survey data were standardised to the German population of 2010, any results of trend analyses cannot be explained by changes in the age-sex composition of the population over time.

### Time trends

Although, evidence underlines the positive health effects of regular physical activity; overall, physicians’ counselling behaviour on physical activity decreased over time. This is in contrast to the increasing evidence about the beneficial effects of physical activity on most chronic non-communicable diseases. Most national and international guidelines for the treatment and prevention of these diseases include physical activity recommendations [2, 8, 10]. Also, due to high or even increasing rates in chronic diseases like diabetes, cancer or cardiovascular diseases and health risk factors like hypertension, physical inactivity or obesity, a 9.4 % prevalence of physical activity counselling in men and 7.7 % in women (2008–2011) seems inadequate to address the major health challenges of an aging society. However, it is positive to note that a trend towards a disease-specific counselling behaviour in terms of a tailored intervention could be observed. Persons with diabetes mellitus or a high cardio-metabolic risk profile reported significant higher counselling proportions in 2008–2011 compared to 1997–1999. This fact could be due to a better availability of disease management and physical activity promotion programmes since 2000 and 2003, respectively [19]. Since that, physicians can refer their patients to structured and quality-assured health promotion programmes. In 2012, solely in the region of North-Rhine with 9.5 million statutory insured persons approximately 500,000 patients with diabetes mellitus were treated in structured disease management programmes [20]. Nevertheless, time trends of about a 10 % increase in physical activity counselling from 2000 to 2010 reported by Barnes et al. from the U.S. and an overall 1/3 of patients being counselled during visits to their doctors in 2010 [15] differ widely from our results. These differences may, at least in part, be explained by differences in the questions asked to study participants. In the National Health Interview Surveys (NHIS) participants were asked whether they had been advised by a physician or other health professional to begin or continue doing exercise or physical activity. In our GNHIES 98 and DEGS 1 studies, participants were asked whether they had received any physical activity counselling provided by a physician. Findings of elevated counselling addressed at persons with diabetes or cardiovascular disease or obesity in the NHIS correspond with our findings, but on a higher level.

### Physical activity promotion

In general, patients’ good accessibility in the primary health care setting is a great opportunity for physicians addressing lifestyle counselling to their patients and can make a contribution to risk factor prevention and the maintenance of a healthy lifestyle. Our results suggest that patients who had been counselled by a physician have at least a two-fold increased likelihood to participate in physical activity promotion programmes compared to uncounselled patients. However, cross-sectional data does not allow making any assumptions on causality.

One reason why many physicians still do not provide information about the health benefits of regular physical activity to their patients may be a lack of time and an insufficient reimbursement for providing counselling services [1, 21]. In 2008 a German health insurance company estimated that a patient’s visit to a doctor takes 7.8 min on average [22]. Under these circumstances comprehensive physical activity recommendations may not be feasible.

Physicians, as an authority on health issues for their patients, have the potential to influence patients’ health by giving them competent advice on physical activity and health behaviour change. As Joy et al. [1] summarised, the “Activity Counseling Trial”, a simple and low cost intervention where physicians’ gave advice and written educational material to their patients, resulted in an increase in physical activity of about 1 kcal/kg/day. This again could be translated in an additional energy expenditure of almost 600 kcal per week in an 80 kg man and in about 25 % lower mortality when compared with those doing less than 500 kcal/week of physical activity [1, 23].

### Limitations

This study has some limitations due its design. Both surveys are part of the ongoing health monitoring system of the Robert Koch Institute. The aim of the monitoring system is to evaluate changes and temporal trends in various health domains on a population-wide basis. Therefore, an extensive interview and examination programme was applied which cannot cover all aspects of healthcare research. However, we feel confident that the data are sufficient to answer our research questions. Unfortunately, the question on physical activity counselling was only asked to persons up to 64 years. This is unfavourable, because many chronic diseases occur in later life and, therefore, the need for preventive interventions like physical activity promotion increases with age. In consequence, our results are not generalizable to the age group of 65 years or older. Secondly, all data on health outcomes, physical activity counselling and programme participation are based on self-reports. For this reason, study outcomes may be affected by recall bias and social desirability. However, we do not feel that this potential bias may influence our results substantially, because the main focus of this paper lies on temporal trends. Therefore, potential bias can be expected to be distributed equally in both surveys.

## Conclusion

Apart from overall low counselling rates provided by primary health care physicians, our results show that patient’s participation in physical activity promotion programmes increased significantly from 1997–1999 to 2008–2011 and is almost doubled in both sexes in the later survey period. This fact could partly be explained by a better availability of physical activity promotion programmes. Since the year 2000, prevention programmes aiming at changes in individual health behaviour, including physical activity, are available to almost anyone and, to a large extent, the costs for these programmes are covered by statutory health insurances [12, 13].

Partially, improved availability of health promotion programmes like structured disease management programmes for patients with chronic diseases like diabetes may have influenced physicians’ counselling behaviour. Therefore, a better availability of physical activity promotion programmes and closer connections to other health professionals like exercise specialists may strengthen the role of prevention and lifestyle intervention in general practice. The fact that men are more likely to be counselled on physical activity behaviour whilst women are more likely to engage in physical activity promotion programmes may point out that existing access strategies for men may need to be reconsidered to make prevention more attractive to men. Furthermore, a special focus of physical activity and health promotion strategies should focus on the reduction of health inequalities. Here, a stronger focus on persons with a low SES should be emphasized. Additionally, training of physicians in brief motivational interviewing could improve their counselling behavior.

Despite only limited resources among physicians (e.g. time, insufficient reimbursement), guiding patients towards an active lifestyle and referring them to physical activity or exercise specialists to take up a supervised exercise programme should be at least a fundamental requirement in physicians’ routine. This would limit the time required by the primary care physician and may increase the number of people counselled to increase physical activity. For example, about 1/3 of more than 90,000 sports clubs in Germany do offer exercise for health programmes to their members at low costs and about 18,000 structured exercise programmes in about 8000 sports clubs are certified by the German Olympic Sports Confederation (DOSB) with the quality seal “Sport for Health”. Currently, a law for health promotion and prevention is being discussed in Germany. The law further integrates physicians in non-medical primary prevention. It is proposed that physicians should make recommendations via a medical certificate to the patient, for example, a prescribed programme of physical activity.

Meanwhile, many different strategies for physical activity counselling and exercise referral were developed. For example, [24] suggested to implement the exercise and physical activity status of every patient as the fifth vital sign on every doctor’s visit [24]. Other strategies already focus on written (“green”) prescription in general practice which should motivate patients to engage in more physical activity [25]. Next to exercise referral interventions, the promotion of self-organised moderate leisure time physical activity like walking or bicycling is another promising approach for physical activity and health improvement for many patients. Additionally, all intervention strategies could be supported by using electronic devices like pedometer, accelerometer or even smartphone applications which count steps or times of physical activity throughout the day.

Considering exercise and physical activity as medicine, further effort should be made to bring physicians, exercise specialists, sport clubs, health insurance companies and policy makers together to achieve sufficient health benefits at the population level and probably save money through reduced health care costs [26].
